# An alkaline and surfactant-tolerant lipase from *Trichoderma lentiforme* ACCC30425 with high application potential in the detergent industry

**DOI:** 10.1186/s13568-018-0618-z

**Published:** 2018-06-05

**Authors:** Yuzhou Wang, Rui Ma, Shigui Li, Mingbo Gong, Bin Yao, Yingguo Bai, Jingang Gu

**Affiliations:** 1 Key Laboratory of Microbial Resources of the Ministry of Agriculture, Institute of Agricultural Resources and Regional Planning, Chinese Academy of Agricultural Sciences, Beijing, 100081 People’s Republic of China; 2 Key Laboratory for Feed Biotechnology of the Ministry of Agriculture, Feed Research Institute, Chinese Academy of Agricultural Sciences, Beijing, 100081 People’s Republic of China

**Keywords:** *Trichoderma lentiforme*, Alkaline lipase, Heterologous expression, Detergent

## Abstract

Alkaline lipases with adaptability to low temperatures and strong surfactant tolerance are favorable for application in the detergent industry. In the present study, a lipase-encoding gene, *TllipA*, was cloned from *Trichoderma lentiforme* ACCC30425 and expressed in *Pichia pastoris* GS115. The purified recombinant *Tl*LipA was found to have optimal activities at 50 °C and pH 9.5 and retain stable over the pH range of 6.0–10.0 and 40 °C and below. When using esters of different lengths as substrates, *Tl*LipA showed preference for the medium length *p*-nitrophenyl octanoate. In comparison to commercial lipases, *Tl*LipA demonstrated higher tolerance to various surfactants (SDS, Tween 20, and Triton X100) and retained more activities after incubation with Triton X100 for up to 24 h. These favorable characteristics make *Tl*LipA prospective as an additive in the detergent industry.
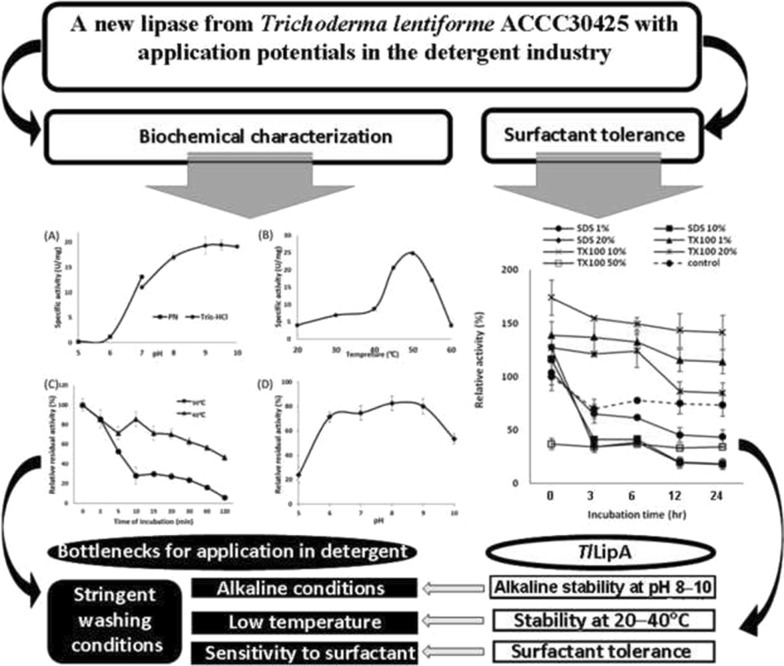

## Introduction

Lipase (EC 3.1.1.3) is regarded as one of the most important commercial enzymes, and has been attracting enormous attention in the rapidly growing biotechnological area. It catalyzes the hydrolysis of triacylglycerols to release diacylglyceride, monoacylglycerol, long-chain fatty acids (> 8 carbons) and glycerol at the interface of oil and water (Brockerhoff 1974). According to the protein structure similarity, lipase belongs to the family of α/β hydrolases, in which a catalytic triad (usually serine, histidine, and aspartic or glutamic acid) and an oxyanion hole (just like a catalytic pocket) are crucial for catalysis (Gupta et al. 2015), and a lid structure involves in the substrate accessibility and binding in the active site (Woolley and Petersen 1996). During hydrolysis, the hydroxy group of the catalytic serine attacks the carbonyl carbon of the ester bond of the substrate, while the catalytic histidine acts as a general-base catalyst and abstracts a proton from the catalytic serine. The alcohol group of the substrate is released and an acyl-enzyme intermediate is formed, which is stabilized in the oxyanion hole by hydrogen bonds. The acyl-enzyme intermediate can be attacked by a water or alcohol molecule, leading to the formation of acid or new ester, respectively (Beer et al. 1996).

Lipases are widespread in nature and have been reported in microbes, plants, and animals. Nevertheless, bacterial and fungal lipases are of special interest as they are easily produced and favorable for industrial purposes due to the high yields and great versatility and stability under harsh conditions (Schmid 2016). Microbial lipases vary in structures and enzymatic properties. For example, the lipases from *Candida rugosa* with different hydrophobic zones at the central channel showed different activities (Mancheño et al. 2003; Domínguez et al. 2006). At the entrance of the channel in close proximity to the catalytic site and the substrate binding site, there is a phenylalanine-rich region associated with substrate binding. The phenylalanine content is negatively correlated with the catalytic activity towards cholesterol ester. In addition, the lipase activity is also related to the size and orientation of the channel. For example, the lipase from *Aspergillus niger* having a narrow and curved channel shows relatively low activity, while those from *Ophiostoma piceae*, *Nectria haematococca* and *Trichoderma reesei* have straightforward and wider channels and much higher activities (Barriuso et al. 2016).

Microbial lipases are widely used in various industries, especially in the detergent (Saxena et al. 2004). Since the 1960s, enzyme-based detergent has been introduced into the market, and lipase that efficiently removes acylglycerols has been one of the major additives in cleaning agent (Abo 1990). The industrial and environmental significances of lipase include but are not limited to: 1 lipase provides an increasable detergency (comparing with the detergent alone), especially at low temperatures and neutral to alkaline pH; 2 lipase with low substrate specificity is highly effective to remove stubborn stains, such as blood and fat; and 3 lipase not only has high biodegradation ability but also brings harmless effect on aquatic ecosystems (Jurado et al. 2007). However, several bottlenecks have limited lipase application in detergent, such as their broad substrate specificity, stringent washing conditions (low temperature and alkaline conditions), and sensitivity to chemicals in detergents (Sharma et al. 2001, 2002). For example, a large number of microbial lipases produced by bacteria and yeast show the maximum activities at high temperatures, such as the lipases from *Pseudomonas aeruginosa*, thermophilic *Bacillus* sp. and yeast *Kurtzmanomyces* sp. that have temperature optima of 60–75 °C. Although some bacterial lipases are neutral to alkaline, but they lose most of the activities at pHs higher than 9.0 (Karadzic et al. 2006; Nawani et al. 2007). Moreover, the sensitivity to surfactant deters numerous lipases from their application in laundry. Therefore, it’s of great value to obtain an alkaline mesophilic lipase with a high tolerance to surfactants in the washing industry (Gutarra et al. 2009).

*Trichoderma* is a common filamentous fungus in soil and root ecosystems, and sometimes in air, water, sand, etc. It shows antagonistic, symbiotic and parasitic capabilities to interact with other microbes and is used more extensively than any other single microbe in agriculture (Benítez et al. 2005). Moreover, *Trichoderma* has significant lignocellulose-degrading capability because it can produce a variety of hydrolytic, lytic and auxiliary enzymes including cellulase, xylanase, chitinase, laccase, lipase, etc. (Zhang and Xia 2017). Besides, *Trichoderma* represents one of the most important expression systems that is widely used to produce industrial enzymes on large scale (Singh et al. 2015). Up to now, the genome sequences of 13 *Trichoderma* strains have been completed (Halliwell and Griffin 1973; Martinez et al. 2008; Kubicek et al. 2011; Studholme et al. 2013; Xie et al. 2014; Baroncelli et al. 2015, 2016; Shikunne et al. 2015; Yang et al. 2015; Lee et al. 2017), and the number keeps increasing. Sequence analysis of the genomes of *T. viride*, *T. reesei*, *T. harzianum* and *T. gamsii* indicated that *Trichoderma* harbors a great variety of lipase genes. In our preliminary studies, four *Trichoderma* strains demonstrated lipase-producing capabilities. One of them, *T. lentiforme* ACCC30425, showed the highest lipase-producing capability under alkaline conditions and thus was selected for draft genome sequencing. In this study, we reported on the gene cloning, heterologous expression, and biochemical characterization of an alkaline mesophilic lipase from *T. lentiforme* ACCC30425. Its application potential as an additive in detergent was assessed as well.

## Materials and methods

### Strains

*Trichoderma lentiforme* ACCC30425 was supplied by the Agricultural Culture Collection of China. *Escherichia coli* Trans1-T1 was purchased from TransGen (China). The heterologous expression system containing the vector pPIC9 and *Pichia pastoris* GS115 competent cells were obtained from the Invitrogen.

### Induction and detection of the lipase production by *T. lentiforme* ACCC30425

*Trichoderma lentiforme* ACCC30425 was grown in the lipase-inducing medium (5 g/L glucose, 5 g/L NaNO_3_, 5 g/L K_2_HPO_4_, 0.3 g/L MgSO_4_, 0.01 g/L FeSO_4_, and 4 g/L olive oil as the sole carbon source) with the agitation rate of 180 rpm at 28 °C for 8 days. The culture supernatants were collected every day and subject to lipase activity assay (spectrophotometric method as described below).

### Cloning of the gene *TllipA*

Seven-day-old mycelia of *T. lentiforme* ACCC30425 were collected, flash-frozen in liquid nitrogen, and ground into a fine powder. Total RNA was extracted using the Trizol method (Chomczynski and Sacchi 1987), and cDNAs were synthesized by reverse transcription. According to the whole genome sequence of *T. lentiforme* ACCC30425 (accomplished by the Majorbio, China), a lipase-encoding gene, *TllipA*, was identified. The nucleotide and amino acid sequences of *TllipA* were analyzed by using the BLASTx and BLASTp programs (https://blast.ncbi.nlm.nih.gov/Blast.cgi), respectively. The signal peptide was predicted using the SignalP 4.0 (http://www.cbs.dtu.dk/services/SignalP/). The prediction of molecular mass and isoelectric point (*p*I) value was performed using the Vector NTI Advance 10.0 software (Invitrogen). Multiple sequence alignment of *Tl*LipA and other lipase representatives was conducted by using the ClustalX 1.81 and presented by ESPript 3.0 (http://espript.ibcp.fr/ESPript/cgi-bin/ESPript.cgi). The putative three-dimensional structure was built by SWISS-MODEL (https://www.swissmodel.expasy.org/) with the lipase from *Ophiostoma piceae* (PDB: 4BE4) as the template.

PCR was then conducted to obtain the DNA fragment coding for mature *Tl*LipA with an expression primer set (*TllipA*-expF: 5′-GGGGAATTCGCTCAAGGCCAAGTCAACGTTACCATTCCC-3′ and *TllipA*-expR: 5′-GGGGCGGCCGCCTAGAAGATCAGTGAATCGATATGCTCCTTGATAAAG-3′, the *Eco*RI/*Not*I sites underlined). The amplification was performed at 94 °C for 5 min followed by 35 cycles of denaturation (1 min at 94 °C), annealing (1 min at 62 °C) and extension (1.5 min at 72 °C), and a final extension of 72 °C for 10 min. The PCR products of the appropriate size were sequenced by Biomed (China).

### Expression of the recombinant *Tl*LipA in *P. pastoris*

The correct PCR products were digested with *Eco*RI and *Not*I and ligated into the *Eco*RI/*Not*I-digested pPIC9 vector to construct the recombinant plasmid pPIC9-*TllipA*. Colony PCR was conducted using the AOX primers to screen positive clones, which were further verified by DNA sequencing. The correct recombinant plasmid was then linearized with *Bgl*II and transformed into *P. pastoris* GS115 competent cells by the electroporation method with the Gene Pulser Xcell Electroporation apparatus (Bio-Rad), following the instructions of Invitrogen’s protocol (2000 V, 200 Ω, 25 μF, and 5 ms). The transformants were grown on minimal dextrose (MD) agar plates at 32°C for 48 h. Ninety-six colonies were randomly selected to grow in 2-mL buffered glycerol complex medium (BMGY) at 30°C for 48 h. The cells were collected by centrifugation (12,000*g*) and resuspended in 2-mL buffered methanol complex medium (BMMY). After 48-h induction with the 0.5% (v/v) methanol at 30°C, the culture supernatants were collected by centrifugation for the lipase activity assay. The positive transformant showing the highest lipase activity was grown in 1-L Erlenmeyer flasks containing 200 mL medium for large-scale fermentation.

### Purification of recombinant *Tl*lipA

The culture supernatants were collected by centrifugation at 12,000*g*, 4°C for 10 min and concentrated through Vivaflow 200 membrane of 5-kDa molecular weight cutoff (Vivascience, Germany). The crude enzyme was loaded onto the HiPrepTm 26/10 Desalting column and HiTrapQHP column (GE Healthcare). Fractions containing lipase activity were pooled and concentrated by ultrafiltration (5-kDa molecular weight cutoff) for further characterization. Sodium dodecyl sulfate-polyacrylamide gel electrophoresis (SDS-PAGE) was carried out with the 5% stacking gel and 12% separation gel. Protein concentration was determined using the Bradford method with bovine serum albumin as the standard.

### Lipase activity assays

The lipase activity was determined by the alkali titration method, using olive oil as the substrate. Olive oil was emulsified in 4% (w/v) polyvinyl alcohol solution at the ratio of 1:3. The reaction mixture contained 2.5 mL of 20 mM citric acid-Na_2_HPO_4_ (pH 7.5), 2.0 mL of emulsified olive oil, and 0.5 mL of properly diluted enzyme solution. After incubation at 40 °C for 15 min in a shaking water bath, 7.5 mL of 95% ethanol was added to terminate the reaction. The amount of liberated fatty acids was measured by titration with 50 mM NaOH, using phenolphthalein as an indicator. One unit (U) of lipase activity was defined as the amount of lipase to liberate 1 μmol of fatty acids per min from the olive oil. All determinations were performed in triplicate.

The spectrophotometric method was also used to determine the lipase activity of purified recombinant *Tl*LipA. The reaction system consisted of 0.1 mL of appropriately diluted enzyme and 2.4 mL of substrate solution containing 0.8 mM *p*-nitrophenyl octanoate (*p*-NPO, C8; dissolved in isopropanol at first) in 20 mM Tris–HCl (specific pH). After incubation at 37 °C (or the optimum temperature of 50 °C) for 15 min, the reactions were terminated by addition of 2.0 mL of 95% ethanol. After centrifugation at 5000*g* for 10 min, 200 μL of each reaction supernatant was transferred to 96-well microplate for absorbance measurement at OD_410_. One unit of lipase activity was defined as the amount of enzyme that produced 1 μmol of *p*-nitrophenol (*p*NP) per min under standard reaction conditions. All determinations were performed in triplicate.

### Effects of pH and temperature on the *Tl*LipA activity

The optimal pH of the recombinant *Tl*LipA was determined at 37 °C for 15 min in the following buffers: 25 mM citric acid-Na_2_HPO_4_ for pH 5.0–7.0 and 20 mM Tris–HCl for pH 7.0–10.0. The optimal temperature was determined over the temperature range of 20–60 °C in 20 mM Tris–HCl (pH 9.5) for 15 min.

### Effects of pH and temperature on the *Tl*LipA stability

The pH stability of *Tl*LipA was determined by measuring the residual lipase activity under the optimal conditions (pH 9.5, 50 °C and 15 min) after pre-incubation of the enzyme at 37 °C for 1 h in the same buffers (pH 5.0–10.0) mentioned above. Thermal stability of *Tl*LipA was determined by measuring the residual lipase activity under the optimal condition after incubation of the enzyme at 40 and 50 °C, respectively, for various periods (0–120 min). The *Tl*LipA activities under optimal conditions (pH 9.5, 50 °C and 15 min) were defined as 100% relative activity.

### Effects of metal ions and chemical reagents on the *Tl*LipA activity

To find out the effects of different metal ions and chemical reagents on *Tl*LipA activity, 5 mM of Na^+^, K^+^, Ca^2+^, Ag^+^, Mg^2+^, Mn^2+^, Zn^2+^, Ni^2+^, EDTA or β-mercaptoethanol was added into the reaction system, respectively. *Tl*LipA activity was determined at pH 9.5 and 50 °C for 15 min with *p*-NPO as the substrate. The *Tl*LipA activities without any chemical addition were defined as 100%.

### Kinetic parameters

*p*-Nitrophenyl esters with different acyl chains (C4–C16) including *p*-nitrophenol butyrate (*p*NPB, C4), *p*NPO (C8), *p*-nitrophenol decanoic acid (*p*NPD, C10), *p*-nitrophenol dodecanoate (*p*NPDD, C12), *p*-nitrophenol myristate (*p*NPM, C14) and *p*-nitrophenol palmitate (*p*NPP, C16) at the concentrations of 0.2–1.6 mM were used as the substrates. The Michaelis–Menten kinetic parameters *K*_*m*_ and *V*_*max*_ of *Tl*LipA were determined at pH 9.5 and 50 °C for 10 min (shorter reaction time for pseudo-first order kinetic analysis). The experiments were repeated for three times, and each experiment included triplicate. GraphPad Prism 5 (GraphPad Software) was used to calculate the *K*_*m*_ (substrate affinity) and *V*_*max*_ (maximum velocity) values. The *k*_*cat*_ (the turnover rate per second) and *k*_*cat*_/*K*_*m*_ (catalytic efficiency) values were then calculated to measure the efficiency of an enzyme that converts substrate to product at sub-saturating substrate concentration and catalytic efficiency (Chinaglia et al. 2014).

### *Tl*LipA tolerance and stability to various surfactants

Surfactant is a common and indispensable ingredient in detergents. To find out the effect of surfactant on *Tl*LipA activity, four concentrations (0.50, 0.20, 0.10 and 0.05%) of Tween 20, Tween 80, Triton X100 or 50 μM SDS (v/v) were individually added into the reaction system containing *p*NPO as the substrate, and the relative activities were tested under optimum reaction conditions (pH 9.5 and 50 °C for 15 min). The reaction systems without any surfactant were treated as controls. To assess the application potential of *Tl*LipA in detergent industry, four commercial lipases (HA, HB, HD and HE) from Xinhuayang Co. (China) were selected as references, and their enzymatic properties were determined as described above.

To assess the stability in the presence of different surfactants, *Tl*LipA was incubated in 20 mM Tris–HCl (pH 9.5) at 37 °C containing 50, 20, 10 or 1% (v/v) of Triton X100, or 20, 10 or 1% (w/v) of SDS for various periods (0, 3, 6, 12, and 24 h). Mesophilic alkaline lipases HA and HB were used as references. The residual activities were determined under the optimum reaction conditions of each enzyme.

## Results

### Olive oil-degrading capability of *T. lentiforme* ACCC30425

**Fig. 1:**
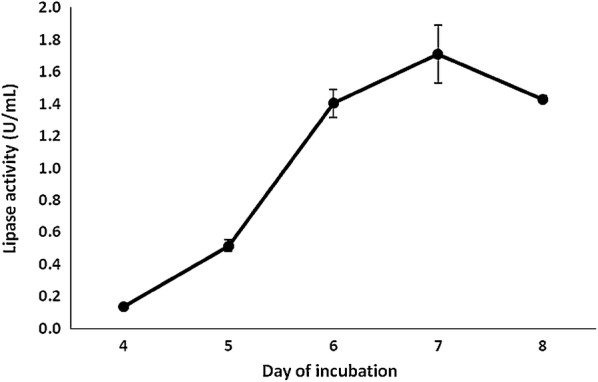
Lipase activities of the culture supernatants of *T. lentiforme* ACCC30425 growing in the inducing medium with olive oil as the sole carbon source. The lipase activities were assayed using spectrophotometric method with *p*NPP as the substrate

By using olive oil as the sole carbon source, *T. lentiforme* ACCC30425 showed detectable lipase activity at day 4 and afterwards (Fig. 1). Using *p*-NPO as the substrate, the lipase activity in the culture supernatants reached maximum at day 7, which was up to 1.7 U/mL (pH 8.0, 37 °C and 15 min). It indicated that *T. lentiforme* ACCC30425 has the capability of producing lipases to degrade olive oil in the medium.

### Sequence analysis of the *TllipA*

**Fig. 2:**
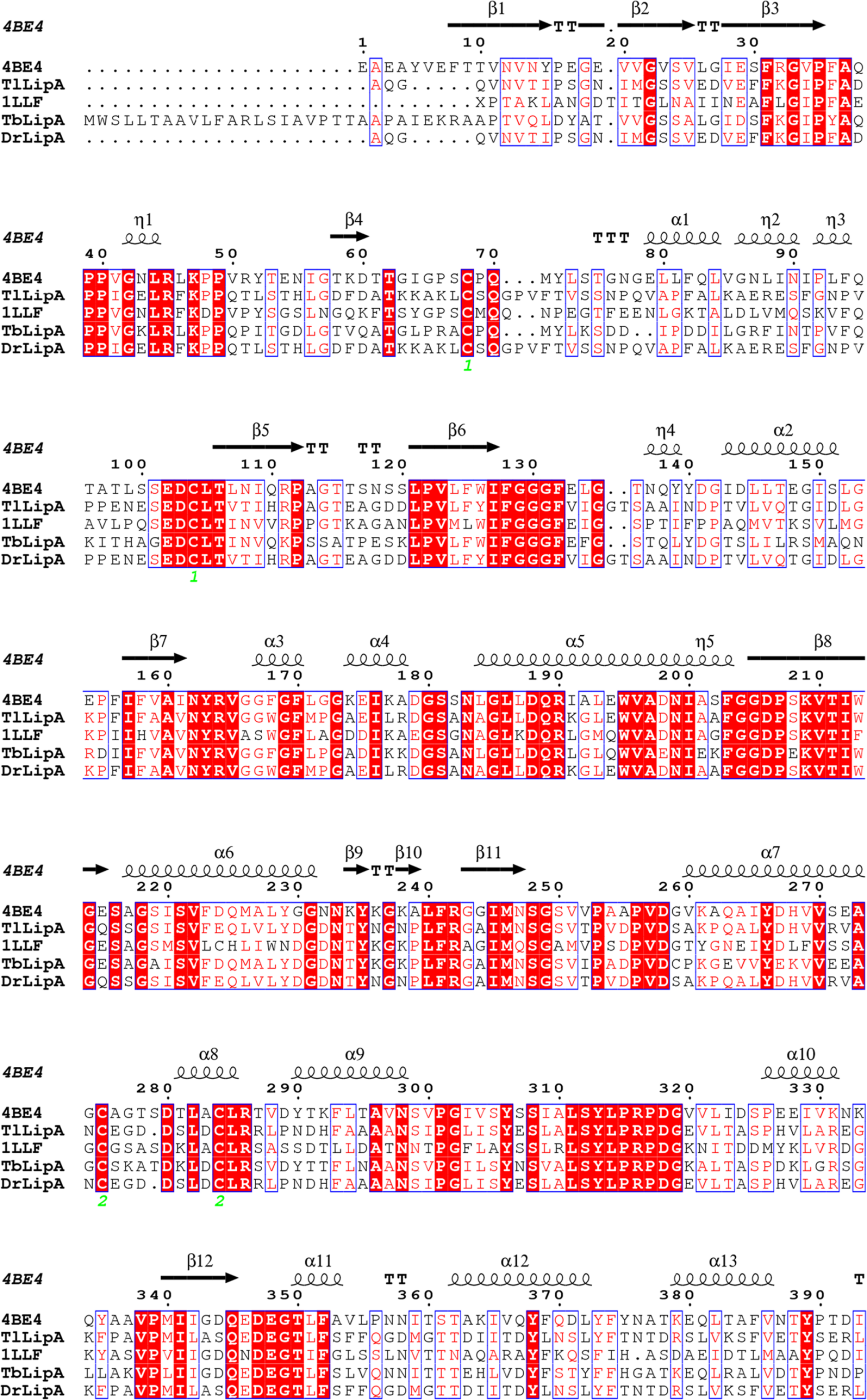
Multiple sequence alignment of deduced *Tl*LipA with structure-resolved lipases 4BE4 from *Ophiostoma piceae* and 1LLF from *Candida cylindracea* as well as biochemically characterized lipases *Tb*LipA from *Trichophyton benhamiae* and *Dr*LipA from *Diutina rugosa*. The secondary structural elements are indicated

**Fig. 3:**
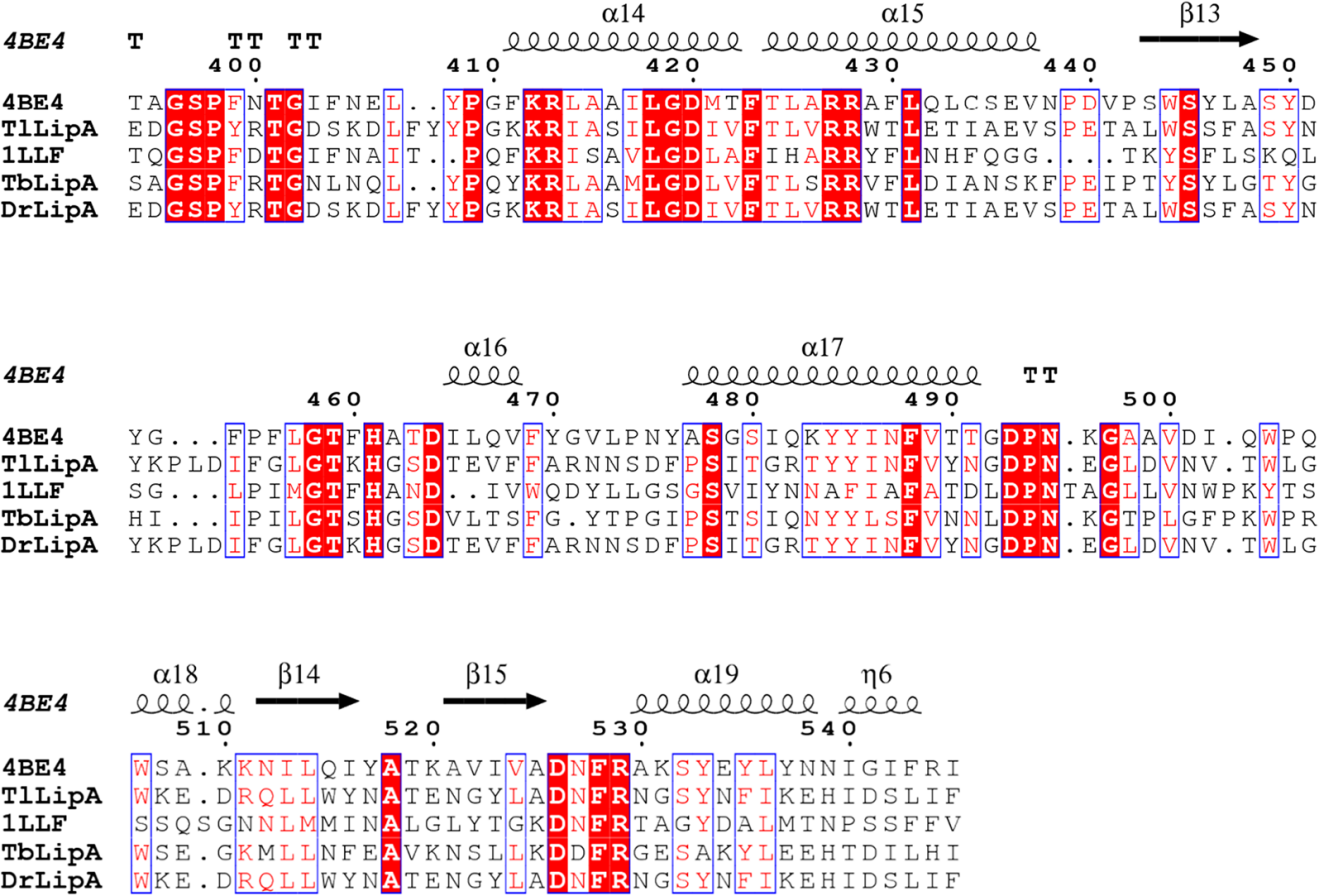
Multiple sequence alignment of deduced *Tl*LipA with structure-resolved lipases 4BE4 from *Ophiostoma piceae* and 1LLF from *Candida cylindracea* as well as biochemically characterized lipases *Tb*LipA from *Trichophyton benhamiae* and *Dr*LipA from *Diutina rugosa*. The secondary structural elements are indicated

Genome sequence analysis indicated that the lipase-encoding gene, *TllipA* (GenBank accession number: MF460438), contains 1707 bp. Deduced *Tl*LipA consists of a putative signal peptide of 20 residues and a mature protein of 548 residues. The molecular mass and *p*I of mature *Tl*LipA were estimated to be 60.0 kDa and 4.56, respectively. Multiple sequence alignment and homology modeling analysis indicated that deduced *Tl*LipA contains the typical α/β-hydrolase fold structure with an N-terminal 3-stranded β-sheet, a major 12-stranded β-sheet, and 19 helices (Fig. 2, 3). The putative catalytic triad consists of S215, E346 and H464. These catalytic residues are responsible for the nucleophilic attack on the carbonyl carbon atom of the ester bond. The putative lid consists of one α-helix (residues 76–81) and two 3_10_-helics (residues 83–87 and 89–91) flanked by two loops that end in a disulfide hinge (residues C62 and C101).

### Production and purification of the recombinant *Tl*LipA

**Fig. 4:**
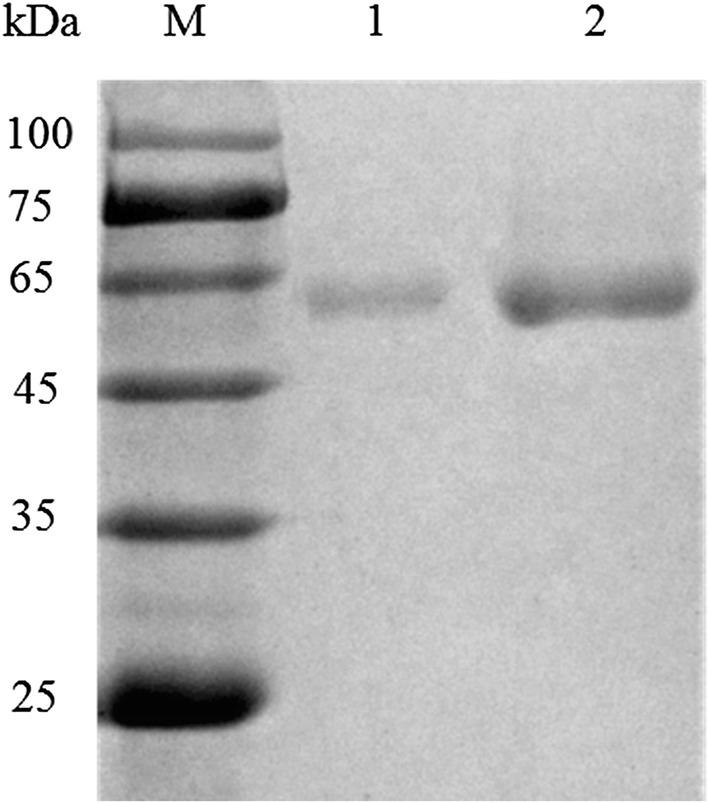
SDS-PAGE analysis of purified recombinant *Tl*LipA. Lane M, the molecular weight standard markers; lane 1, the purified recombinant *Tl*LipA at 0.3 mg/mL; and lane 2, the purified recombinant *Tl*LipA at 3.0 mg/mL

The DNA fragment coding for the mature *Tl*LipA was obtained with primers *TllipA*-expF and *TllipA*-expR, and transformed into *E. coli* Trans1-T1 for sequencing. The correct PCR product was digested with *Eco*RI and *Not*I and then cloned into the pPIC9 vector in-frame fusion of the α-factor signal peptide to construct the recombinant plasmid pPIC9-*TllipA*. The recombinant plasmid was linearized by *Bgl*II, and transformed into the *P. pastoris* GS115 competent cells by electroporation. *Tl*LipA was successfully produced according to the *Pichia* expression kit and secreted into the culture. After centrifugation, concentration and exchange chromatography, the crude enzyme was purified to electrophoretic homogeneity, showing a single band of approximately 60 kDa in SDS-PAGE (Fig. 4). This molecular mass was similar to the theoretical value (60.0 kDa), indicating that the band was purified recombinant *Tl*LipA indeed. The lipase activity of purified recombinant *Tl*LipA was determined to be 10.4 ± 0.5 U/mL by using the alkali titration method.

### Effect of pH and temperature on *Tl*LipA activity

**Fig. 5:**
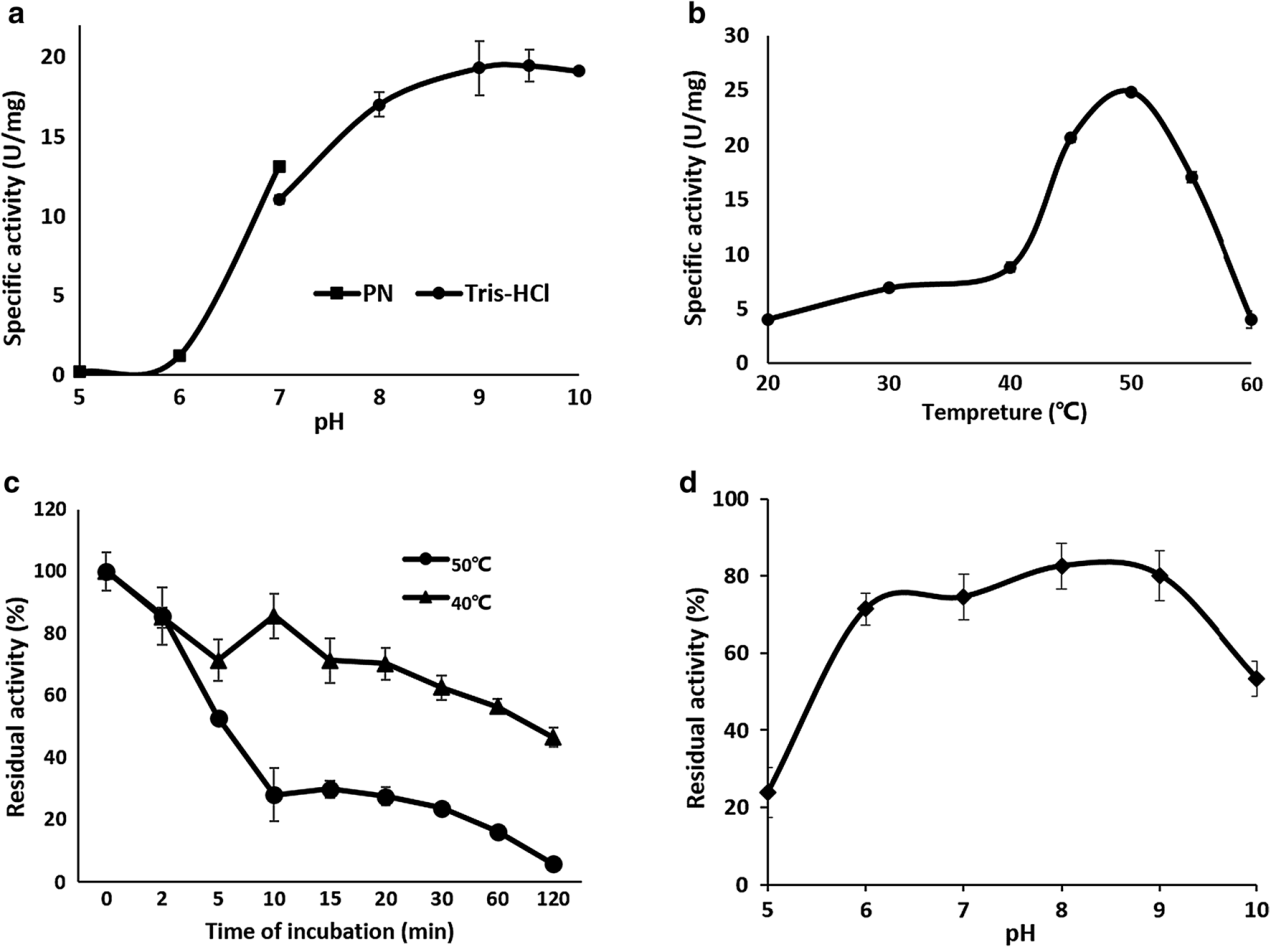
Biochemical characterization of *Tl*LipA. **a** The pH-activity profiles determined in citric acid-Na_2_HPO_4_ (black square) and Tris–HCl (black circle) buffers. **b** The temperature-activity profile determined in Tris–HCl buffer at pH 9.5. **c** The thermal stability at 50 °C (black circle) and 40 °C (blackup-pointing triangle). **d** The pH stability after 1-h incubation at 37 °C and pH 5.0–10.0

*p*NPO was used as the substrate for biochemical characterization of purified recombinant *Tl*LipA. Over the range of pH 5.0–10.0, the *Tl*LipA had poor activity under acidic conditions and showed maximum activity at pH 9.5 (Fig. 5a). Under the optimum pH (9.5), the temperature-activity profile of *Tl*LipA was determined over the temperature range from 20 to 60 °C. The enzyme had a temperature optimum at 50 °C and remained 20–40% of the maximum activity at 20–40 °C (Fig. 5b). These results indicated that the purified recombinant *Tl*LipA is a mesophilic alkaline lipase with great adaptation to low moderate temperature.

### Thermal and pH stability of *Tl*LipA

After incubation at 40 and 50 °C respectively for various periods, aliquots of *Tl*LipA were withdrawn for residual activity assay. As shown in Fig. 5c, *Tl*LipA was relatively stable at 40 °C, retaining more than 60% of the initial activity after 60-min incubation; when extended to 120 min, more than 50% activity was still retained. In contrast, it lost stability at 50 °C, losing more than 50% activity within 5-min incubation and almost all activity within 120-min. The pH stability of *Tl*LipA was also assessed. The enzyme was stable at pH 6.0–9.0, retaining more than 80% of the initial activity after 60-min pre-incubation at 37 °C (Fig. 5d). These results indicated that *Tl*LipA was stable over the cold (≤ 40 °C) and neutral to alkaline conditions.

### Kinetic parameters

The kinetic parameters *K*_*m*_, *V*_*max*_, *k*_*cat*_ and *k*_*cat*_*/k*_*m*_ of *Tl*LipA were determined using the six *p*-nitrophenyl esters of various acyl chain lengths as substrates. As shown in Table 1, *Tl*LipA showed higher affinities (decreased *K*_*m*_ values) and catalytic efficiencies (increased *k*_*cat*_*/k*_*m*_ values) towards short-chain substrates with (C4 to C10). *p*NPO (C8) as the preferred substrate was catalyzed with the highest efficiency of 41.0/s mM.

**Table 1:** The kinetic values of purified recombinant *Tl*LipA towards esters of different lengths

Substrate	*V*_*max*_ (μmol/min mg)	*k*_*cat*_ (/s)	*K*_*m*_ (mM)	*k*_*cat*_*/K*_*m*_ (/s mM)
*p*NPO (C8)	27.3 ± 4.4	27.4 ± 4.4	0.67 ± 0.23	41.0
*p*NPD (C10)	12.5 ± 0.7	12.5 ± 0.7	0.35 ± 0.07	35.8
*p*NPB (C4)	12.2 ± 0.8	12.2 ± 0.8	0.40 ± 0.07	30.7
*p*NPDD (C12)	16.8 ± 2.4	16.8 ± 2.4	1.59 ± 0.42	10.6
*p*NPM (C14)	4.3 ± 1.0	4.3 ± 1.0	3.29 ± 1.21	1.32
*p*NPP (C16)	4.8 ± 1.0	4.8 ± 1.0	4.22 ± 1.28	1.14

### Effect of metal ions and chemical reagents on *Tl*LipA activity

Of the ten chemicals tested in this study (Table 2), Ni^2+^, Zn^2+^, Mn^2+^ and EDTA strongly inhibited the *Tl*LipA activity, leading to the activity loss of more than 50%, while other chemicals had no or little effects (0–32%). None of the chemical addition resulted in an improvement of lipase activity. The results indicated that *Tl*LipA was tolerant to most tested metal ions and chemical reagents.

**Table 2:** Effects of metal ions and chemical reagents on the *Tl*LipA activity

Chemicals	Relative activity (%)^a^	Chemical	Relative activity (%)
CK	100.0 ± 2.4	Mn^2+^	13.9 ± 0.8
Mg^2+^	97.6 ± 2.5	Zn^2+^	11.9 ± 0.9
Ca^2+^	92.4 ± 4.0	Ni^2+^	7.6 ± 0.8
K^+^	91.1 ± 3.0	β-Mercaptoethanol	60.5 ± 0.9
Na^+^	85.7 ± 6.8	EDTA	33.8 ± 2.0
Ag^+^	68.6 ± 3.8		

^a^Values are given as the mean ± standard deviations (n = 3)

### *Tl*LipA tolerance to surfactants

**Fig. 6:**
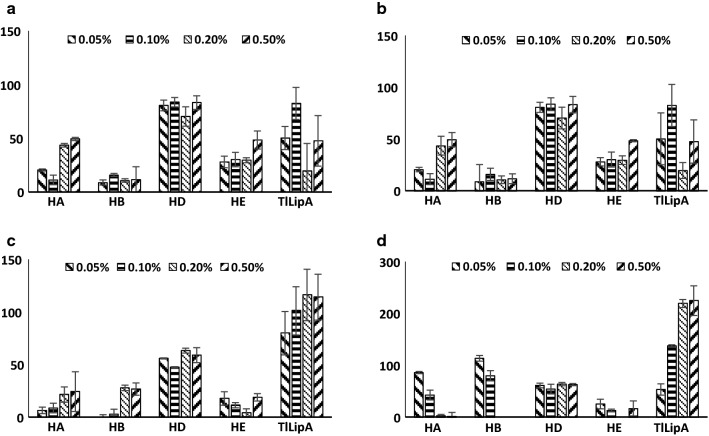
Tolerance of *Tl*LipA and four commercial lipases HA, HB, HD and HE to Tween 20 (**a**), Tween 80 (**b**), Triton X100 (**c**) and SDS (**d**) at different concentrations, respectively

To find out the application potentials of *Tl*LipA in detergent industry, we selected four commercial lipases as references and compared their activities with *Tl*LipA in the presence of 0.05–0.50% of Tween 20, Tween 80, Triton X100 or SDS. As shown in Fig. 6, Tween 20, SDS and Triton X100 at higher concentrations (0.10–0.50%) significantly enhanced the *Tl*LipA activity up to 2.24-fold, while 0.05–0.50% of Tween 80 and 0.05% of Tween 20, SDS and Triton X100 inhibited the lipase activity of *Tl*LipA by 20‒60%. It indicated that high concentrations of Tween 20, Triton X100 and SDS may emulsify the substrate to enlarge the interface area between *Tl*LipA and substrate, consequently improving the lipase activities. On the contrary, the activities of the four commercial lipases were mostly inhibited by the surfactants under each optimum condition, respectively (pH 9.0 and 50 °C for HA and HB, pH 8.0 and 50 °C for HD, and pH 8.0 and 40 °C for HE), with the only exception of increased HB activity (approximately 13.4%) by 0.05% SDS. The results indicated that *Tl*LipA was highly tolerant to all tested surfactants and retained most or even enhanced activities.

### *Tl*LipA stability to surfactants

**Fig. 7:**
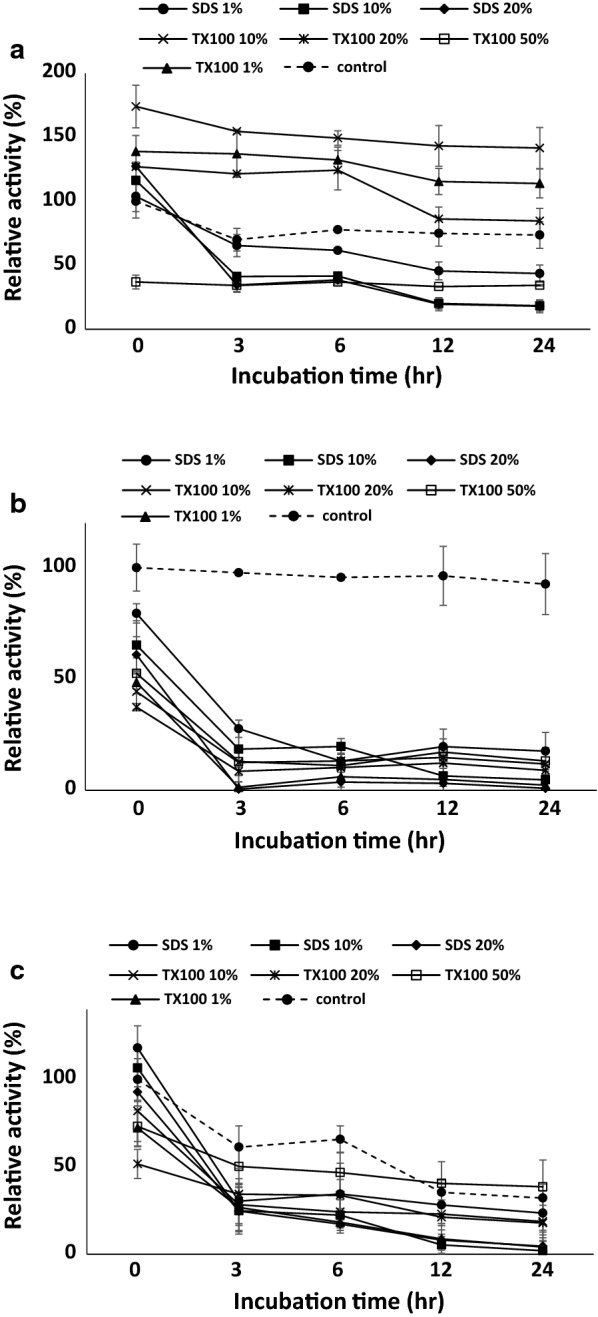
Stability of *Tl*LipA (**a**) and commercial lipases HA (**b**) and HB (**c**) after incubation with SDS or Triton X100 for various durations

Considering the long shelf life of laundry detergent, lipase stability in the presence of surfactants is a key factor of commercialization. Therefore we also determined the *Tl*LipA stability after pre-incubation with 1–20% SDS or 1–50% Triton X100 for various periods. In comparison to the surfactant-untreated controls, *Tl*LipA incubated with 1‒20% Triton X100 for 0–24 h showed significantly enhanced activities of 1.3‒1.7-fold, but lost more than 50% activity when incubated with 50% of Triton X100 and 1‒50% SDS (Fig. 7a). In contrast, both HA and HB lost stability in the presence of 1‒50% of Triton X100 or SDS (Fig. 7b, c), retaining less than 30% activity after incubation at 3 h.

## Discussion

The genus *Trichoderma* contains a very large group of important microorganisms. It is not only a genetic resource of various functional proteins (Freitas et al. 2014) but also a key workhorse for enzyme production on commercial scale (i.e. *T. reesei*) (Jørgensen et al. 2014). In the present study, we reported an alkaline, mesophilic lipase-producing strain (ACCC30425) of *T. lentiforme*. Along with the rapid progress of genome sequencing (Yang et al. 2015), to obtain objective genes with special characters is very simple and efficient. Based on the sequence analysis and annotation of the genome of *T. lentiforme* ACCC30425, the full-length *TllipA* was identified and its structure and functions were predicted. Although *TllipA* shows high sequence identity (100%) to the hypotheoretical lipase from *T. guizhouense*, its identities to lipases with function verified or structure resolved are much lower (< 50%). Thus it is of importance and novelty to clone the gene and produce the gene product for potential applications in various industries.

Most fungal lipases act over a broad pH range, with the pH optima of 4.0–8.0 (Sharma et al. 2011; Singh and Mukhopadhyay 2012), and are mesophilic with thermolability at > 40 °C (Gutarra et al. 2009). The pH optimum of *Tl*LipA was 9.5, which is higher than most fungal lipases characterized so far. Moreover, it showed great adaptability and stability under neutral to alkaline conditions (pH 7.0–10.0). On the other hand, *Tl*LipA showed maximum activity at 50 °C and thermolability at > 40 °C. These enzymatic properties make *Tl*LipA potential for application in the alkaline and low to moderate temperature fields, especially the washing industry (Jurado et al. 2007; Grbavčić et al. 2011). Some lipases are resistant to heavy metals, such as Ca^2+^, Fe^2+^, and Mg^2+^ (Jurado et al. 2007; Gaur et al. 2008; Rao et al. 2009; Sethi et al. 2016). *Tl*LipA showed similar tolerance to Ca^2+^ and Mg^2+^, but showed sensitivity to heavy metals Ni^2+^, Mn^2+^ and Zn^2+^ and chemical reagents EDTA and β-mercaptoethanol. Thus the effects of metal ions and chemical reagents on *Tl*LipA should be considered for future application.

Lipase is usually considered as an enzyme that hydrolyzes the cleavage of long-chain acylglycerols; however, most known lipases are also active on shorter acyl chain esters (Li and Zong 2010). *Tl*LipA having lipase activity as determined by the alkali titration method is a true lipase, but it prefers medium-chain fatty acid esters. The catalytic efficiencies (*k*_*cat*_*/K*_*m*_) of *Tl*LipA towards *p*NPO (C8) and *p*NPD (C10) were higher than that towards other esters of different lengths. Similar results had been reported on the two highly thermophilic alkaline lipases from *Thermosyntropha lipolytica* (Moh’d and Wiegel 2007).

The resistance to surfactant is a big challenge for lipase’s application in washing industry. In general, the surfactant has negative effects on enzymatic hydrolysis and represents a competitive inhibiter in the reaction system (Tatara et al. 1985). *Tl*LipA remained highly active in the presence of both anionic and non-ionic surfactants, including SDS, Triton X100, Tween 20 and Tween 80. Moreover, the resistance of *Tl*LipA to surfactants showed improvement along with increased concentration. For example, when increased the concentration of SDS from 0.05 to 0.50%, the relative activity was enhanced from 60 to 224%. This result is contrary to the commercial lipases tested in this study and the previous study that the lipase activity would drop down along with the increased concentration of SDS (Rathi et al. 2001). Moreover, incubation with the increased concentrations of Triton X100 simulated the *Tl*LipA activities but strongly inhibited the enzymatic activities of commercial lipases HA and HB. In comparison to the stabilities of other lipases with surfactants (1-h incubation) (Rathi et al. 2001; Bora and Kalita 2010), *Tl*LipA retained similar or higher activity in the presence of 10% Triton X100 even over 24 h-incubation (150% vs. 93‒164%). Thus the alkaline mesophilic *Tl*LipA with adaptability and stability to broad pH and temperature ranges and high tolerance to surfactants is favorable for potential application in the washing industry.

## Abbreviations

BMGY: buffered glycerol-complex medium
BMMY: buffered methanol-complex medium
MD: minimal dextrose
PAGE: polyacrylamide gel electrophoresis
SDS: sodium dodecyl sulfonate
pNP: *p*-Nitrophenol
pNPB: *p*-Nitrophenol butyrate
pNPD: *p*-Nitrophenol decanoic acid
pNPDD: *p*-nitrophenol dodecanoate
pNPM: *p*-Nitrophenol myristate
pNPO: *p*-Nitrophenyl octanoate
pNPP: *p*-Nitrophenol palmitate
*p*I: isoelectric point
